# Hallermann–Streiff syndrome: A systematic review of the published literature

**DOI:** 10.1016/j.jobcr.2026.101485

**Published:** 2026-06-24

**Authors:** Rezhat Abbas, Revathi Krishna, Aarushi Garg

**Affiliations:** Department of Oral & Maxillofacial Pathology and Microbiology, Maulana Azad Institute of Dental Sciences, BSZ Marg, New Delhi, 110002, India

**Keywords:** Hallermann–Streiff syndrome, Oculomandibulodyscephaly, Craniofacial anomalies

## Abstract

**Background:**

Hallermann–Streiff syndrome is an extremely rare congenital disorder characterized by craniofacial dysmorphism, ocular abnormalities, hypotrichosis, and mandibular hypoplasia, frequently associated with complex airway challenges. No prior systematic review has comprehensively evaluated airway, ophthalmologic, and craniofacial manifestations together. This review synthesizes clinical phenotypes, management strategies, and outcomes from published cases.

**Methods:**

A systematic search of PubMed, Scopus, Embase, and ScienceDirect identified 42 records following PRISMA 2020 guidelines. After screening, 15 studies comprising 32 cases were included. Extracted data included demographics, airway involvement, ophthalmologic findings, craniofacial/orodental anomalies, interventions, and outcomes. Risk of bias was assessed using the JBI checklist for case reports.

**Results:**

Airway involvement was reported in over half of cases, ranging from mild obstruction to life-threatening respiratory compromise requiring tracheostomy or specialized anesthetic management. Ophthalmologic abnormalities were common, including congenital cataracts (76%), microphthalmia (65%), and strabismus (59%). Craniofacial anomalies such as micrognathia (88%) and mandibular hypoplasia (82%) were consistently observed. One report documented three-generation familial inheritance. Interventions were multidisciplinary, with most cases showing partial improvement, although severe neonatal respiratory failure remained fatal.

**Conclusions:**

Hallermann–Streiff syndrome presents with a characteristic triad of craniofacial, airway, and ophthalmologic abnormalities requiring coordinated multidisciplinary care and long-term follow-up.

## 1 Introduction

Hallermann–Streiff syndrome is an uncommon congenital condition originally identified as oculomandibulodyscephaly, first documented by Hallermann in 1948 and later further characterized by Streiff in 1950. Although the precise etiology remains uncertain, current evidence suggests that the disorder may arise from a de novo genetic alteration, with particular suspicion directed toward variants in GJA1, the gene encoding the gap-junction protein connexin-43.^1^ (see Table 5)

François outlined seven key clinical features that serve as diagnostic hallmarks of Hallermann–Streiff syndrome. These include dyscephaly with a characteristic bird-like facial appearance, dental anomalies, proportionate short stature, hypotrichosis, and cutaneous atrophy predominantly affecting the nasal region, bilateral microphthalmia, and congenital cataracts.^2^ The condition remains exceedingly rare, with the available literature consisting predominantly of isolated case reports and small case series scattered across decades. This fragmented evidence base limits a comprehensive understanding of the syndrome's phenotypic spectrum, ophthalmologic and craniofacial manifestations, airway implications, and management challenges. The absence of consolidated data also hampers early diagnosis and informed clinical decision-making. Therefore, a systematic synthesis of all published cases is essential to collate the diverse clinical features, identify consistent patterns, highlight gaps in knowledge, and support clinicians in recognizing and managing this rare disorder more effectively.

## 2 Methods

### 2.1 Information sources and search strategy

This review was conducted and reported in accordance with the PRISMA (Preferred Reporting Items for Systematic Reviews and Meta-Analyses) guidelines. It was registered with the International Prospective Register of Systematic Reviews (PROSPERO) database under registration number CRD420251248477, ensuring methodological transparency and adherence to best practices in systematic review conduct. A comprehensive literature search was conducted on 30th November, 2025 across PubMed/MEDLINE, Scopus, Embase, and ScienceDirect. Reference lists of the included studies were not manually screened, and no additional search strategies beyond the predefined database search were employed. The search strategy incorporated both controlled vocabulary and free-text terms to identify all relevant publications on Hallermann–Streiff syndrome. The search string used across databases was:

("Hallermann-Streiff syndrome" OR "Hallermann Streiff" OR "Hallermann–Streiff" OR "oculomandibulodyscephaly" OR "HSS") AND (case report OR case series OR clinical features OR diagnosis).

This strategy was adapted for each database according to its specific search syntax. No restrictions were applied regarding language, publication year, or study design to ensure maximal completeness. All search results were imported into reference management software, and duplicate records were removed prior to screening.

### 2.2 Data extraction

Data extraction was carried out independently by two reviewers (RA and RK) using a pre-structured and standardized data collection form specifically designed for case reports and case series. Relevant information extracted from each included study comprised patient demographics, clinical presentation, craniofacial and orofacial manifestations, ophthalmologic findings, airway characteristics, management strategies, and follow-up outcomes.

In instances of ambiguous or incomplete data, interpretation was based strictly on the explicit details provided within the original reports. Any discrepancies between the two reviewers were resolved through discussion until consensus was achieved. The extracted data were systematically compiled and organized into detailed summary tables to facilitate qualitative synthesis.

#### 2.2.1 Inclusion criteria

• Published case reports and case series focusing on the condition of interest
• Studies providing sufficient clinical, diagnostic, and/or management details
• Articles available in full text and in English

#### 2.2.2 Exclusion criteria

• Review articles, editorials, and letters without primary patient data
• Studies lacking adequate clinical details
• Duplicate publications or overlapping datasets

### 2.3 Risk of bias assessment

The methodological quality of all included case reports was assessed using the Joanna Briggs Institute (JBI) Critical Appraisal Checklist for Case Reports. Each study was evaluated independently by two reviewers across the eight JBI domains, including clarity of patient history, diagnostic tests, intervention details, post-intervention outcomes, adverse events, and takeaway lessons. Discrepancies between reviewers were resolved through discussion and consensus. The overall risk of bias for each report was categorized based on the proportion of criteria met, and findings from this appraisal were incorporated into the interpretation of results.

## 3 Results

### 3.1 Study selection

A total of 42 records were identified through electronic database searching (PubMed = 8, Scopus = 14, Embase = 8, and ScienceDirect = 12). After removing 12 duplicate records, 27 articles remained for title and abstract screening. Of these, 10 records were excluded (5 review articles and 5 with an inappropriate study design).

**Fig. 1:**
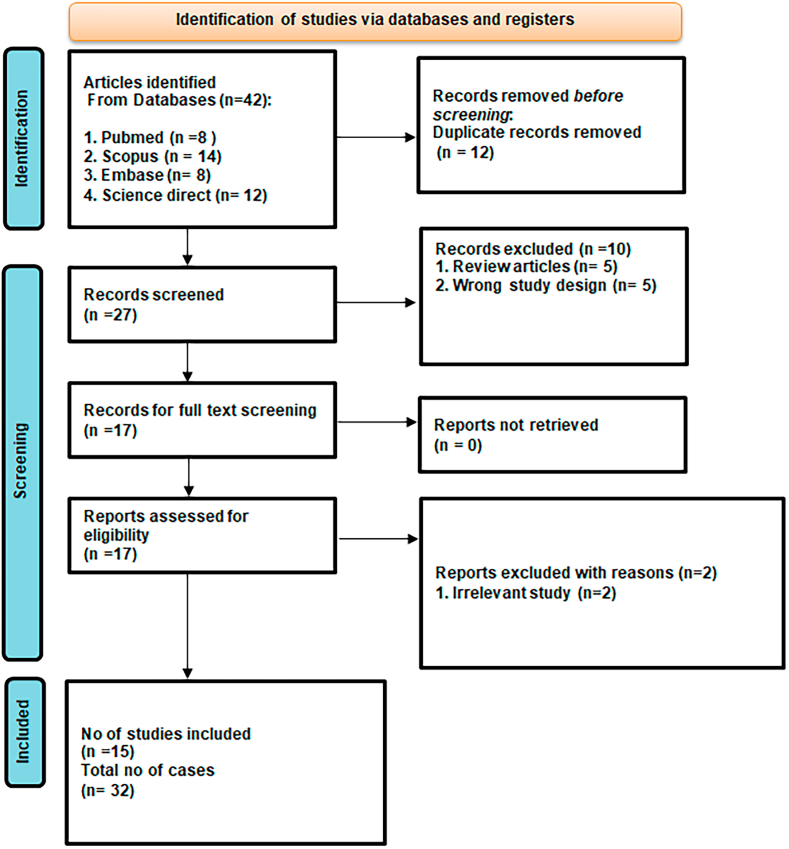
PRISMA Flow Diagram showing records identified, screened, assessed for eligibility, and included in the review.

A total of 17 full-text articles were assessed for eligibility. No articles were unavailable for retrieval. Following full-text evaluation, 2 reports were excluded because they did not meet the inclusion criteria. Finally, 15 studies, reporting a total of 32 individual cases of Hallermann–Streiff syndrome, were included in the qualitative synthesis (Fig. 1).

### 3.2 Study characteristics

A total of 15 studies encompassing 32 individual cases of Hallermann–Streiff syndrome (HSS) were included in this review. The publications ranged from 1991 to 2024 and originated from diverse geographical regions including Europe, Asia, Africa, and the Americas, highlighting the global distribution of reported cases.

Patient ages showed wide variability, ranging from newborns to 77 years, with cases reported across all age groups. Most cases involved female patients, although both sexes were represented. The clinical presentations demonstrated significant heterogeneity, with airway-related manifestations documented in a substantial proportion of reports. These ranged from mild breathing difficulties to life-threatening upper airway obstruction, severe obstructive sleep apnea, and tracheomalacia, while some cases reported no airway involvement.

Management strategies varied depending on severity and organ involvement. Interventions included orthognathic and dental procedures, ophthalmologic surgeries (particularly for congenital cataracts), non-invasive ventilation, adenoidectomy, gastrostomy, lymphatic therapy, and multidisciplinary supportive care. Airway management challenges were highlighted in several studies, including difficult intubation and the need for tracheostomy in select cases. Many cases, however, were managed conservatively with observation and routine monitoring.

Follow-up duration varied considerably across studies, from short-term postoperative review to longitudinal monitoring extending up to several years. Some cases reported stable outcomes, while others—particularly those with severe airway compromise—had poor prognoses, including mortality secondary to respiratory complications (Table 1).

**Table 1:** Study characteristics.

S.no	Author, Year	Country	Number of Cases	Age	Sex	Airway Findings	Interventions/Management	Follow-up
1	Sondeijker C.F.W et al. 2021^13^	Netherlands	1	53 years	Female	Severe obstructive sleep apnea	Mandibular repositioning appliance, fixed orthodontics, extractions, bimaxillary orthognathic surgery	2-year follow-up with multiple intervals
2	Preudhomme R. et al. 2022^10^	France	Case 1	3 years	Not specified	Breathing difficulties	Conservative management; planned monitoring; lipofilling of nose region.	NR
			Case 2	10 years	Not specified	Breathing difficulties	Lipofilling for nasal abnormalities; skin management; ophthalmologic care	NR
3	Abdulghafor M. et al. 2024^9^	Switzerland	1	10-day-old neonate	Female	Severe obstructive sleep apnea at 9 months, feeding issues.	Bilateral lensectomy, posterior capsulorhexis, anterior vitrectomy, semirigid contact lens correction, nocturnal NIV for OSA, growth hormone therapy, gastrostomy feeding	4.5-year follow-up with progressive visual monitoring
4	Robinow M et al. 1991^16^	USA	1	4 months	Male	Severe upper airway obstruction from birth; inability to breathe through nose; recurrent aspiration pneumonias; progressive cor pulmonale; eventual respiratory failure	Feeding gastrostomy at 6 months, antibiotics, supportive care; no airway surgery; aspiration pneumonia management	Died at 11 months; recurrent aspiration, *H. influenzae* meningitis; worsening cor pulmonale
5	Pereira de Godoy AC et al. 2022^17^	Brazil	1	18 years	Female	Lifelong nasal obstruction, unable to breathe through nose.	Cervical lymphatic therapy, linear facial lymphatic drainage, mechanical lymphatic therapy, compression therapy	Breathing improvement sustained for months; 2-year treatment course; obstruction recurs but resolves after single lymphatic session
6	Jimenez-Armijo A. et al. 2021^5^	Morocco	1	3-year-old	Female	Obstructive sleep apnea syndrome	Dental extractions for abscesses, preventive dental care, removable denture at age 6.	2+ years of follow-up (3 to 6 years)
7	Shimada A. et al. 2022^15^	Japan	1	68 years	Female	No airway symptoms or obstruction reported	Cataract surgery + Goniosynechialysis OD; EDTA chelation for corneal calcification OS; Tissue plasminogen activator; Membranectomy.	16 months – improved BCVA OD; stable IOP without medication
8	Guerin S. et al. 2022^14^	Switzerland	1	Infant – 38 weeks	Female	Severe obstructive sleep apnea	Adenoidectomy, Gastrostomy, CPAP trial, BPAP NIV successful	2-year follow-up; stable respiratory status with NIV, developmental improvement, no major adverse events
9	Agrawal P et al. 2021^3^	India	1	21 years	Female	difficult airway	Ventilated with paediatric mask; Intubation with bougie in second attempt; Standard GA with sevoflurane, vecuronium.	Successful surgery; Uneventful recovery; Immediate post-op period stable
10	Neki AS 1993^7^	India	1	50 years	Female	NR	NR	NR
11	David LR et al. 1999^12^	USA	15	32 years (mean age)	Male:7	Respiratory difficulties (11 cases)	Tracheostomy (3 cases)	1 death reported
					Female:8			
12	Epée E et al. 2017^4^	Cameroon	Case 1, father	77 years	Male	NR	Cataract management only	Routine ophthalmologic follow-up
			Case 2, Daughter	39 years	Female	NR	Ophthalmologic management;	Standard ophthalmology follow-up
			Case 3, grand daughter	19 years	Female	NR	Ophthalmologic management;	Ophthalmology follow-up
13	Jain V et al., 2011^8^	India	1	15 years	Female	Small nares; glossoptosis; tracheomalacia; narrow airway passage; abnormal glottic closure; history of recurrent respiratory infections	ENT, ophthalmology, gynecology referrals; dental treatment	Regular dental visits; periodic ENT & ophthalmology follow-up;
14	Ammar N et al. 2022^6^	Turkey	1	5 years	Female	Respiratory distress in neonatal period (45-day NICU stay).	Dental procedures performed + behavioral management.	- Regular monthly follow-up for 3 months, then every 3 months. - No airway complications reported during follow-up.
15	Lee MC et al. 2008^11^	Korea	1	5 years 7months	Female	Stridor during sleep	Ophthalmologic surgeries for cataracts	- Postoperative ophthalmologic follow-up with glasses prescribed

Overall, the included studies underscore the clinical diversity of HSS, particularly regarding airway involvement, and emphasize the importance of individualized, multidisciplinary management.

### 3.3 Craniofacial and orofacial features

Table 2 summarizes the major craniofacial and orofacial characteristics reported across the included case reports of Hallermann–Streiff syndrome. Analysis of the 32 reported cases demonstrated a consistent pattern of craniofacial involvement characteristic of Hallermann–Streiff syndrome. Micrognathia emerged as one of the most frequently reported findings, present in the majority of cases across all age groups and geographic regions. Mandibular hypoplasia and pinched or narrow nasal morphology were also highly prevalent, reinforcing the classical “bird-like facies” associated with the syndrome. In several cases—particularly those reported by Agrawal et al.^3^, Ammar et al.^16^., and Epée et al.^4^—this composite facial appearance was explicitly described.

**Table 2:** Craniofacial and Orofacial characteristics reported across the included case reports of Hallermann–Streiff syndrome.

S. no	Author	Micrognathia	Microstomia	Retrognathia	Pinched Nose	Dental Anomalies	Enamel Fragility	High-Arched Palate	Bird-like Facies	Hypotrichosis/Alopecia	Mandibular Hypoplasia	Frontal Bossing
1	Sondeijker C.F.W et al. 2021^13^	Yes	No	Yes	Yes	Yes	No	No	No	No	Yes	Yes
2	Preudhomme R. et al. 2022^10^ (Case 1)	Yes	Yes	No	No	Yes	No	No	No	Yes	Yes	Yes
3	Preudhomme R. et al. 2022^10^ (Case 2)	Yes	Yes	No	Yes	No	No	No	No	No	Yes	No
4	Abdulghafor M. et al. 2024^9^	Yes	No	No	Yes	Yes	No	No	Yes	No	Yes	Yes
5	Robinow M et al. 1991^16^	Yes	Yes	No	Yes	Yes	No	No	No	No	Yes	No
6	Pereira de Godoy AC et al. 2022^17^	Yes	No	No	Yes	Yes	No	No	No	No	No	No
7	Jimenez-Armijo A. et al. 2021^5^	No	Yes	Yes	Yes	Yes	Yes	Yes	No	Yes	Yes	Yes
8	Shimada A. et al. 2022^15^	Yes	No	No	No	Yes	No	No	No	No	No	No
9	Guerin S. et al. 2022^14^	Yes	No	Yes	Yes	Yes	No	No	No	No	No	No
10	Agrawal P et al. 2021^3^	Yes	Yes	No	Yes	No	No	No	Yes	No	Yes	Yes
11	Neki AS 1993^7^	Yes	NR	NR	Yes	NR	NR	NR	Yes	Yes	Yes	NR
12	Jain V et al., 2011^8^	No	No	No	Yes	Yes	No	No	Yes	Yes	No	Yes
13	Ammar N et al. 2022^6^	Yes	Yes	Yes	Yes	Yes	Yes	Yes	Yes	Yes	Yes	Yes
14	Lee MC et al. 2008^11^	Yes	NR	NR	Yes	Yes	NR	NR	Yes	Yes	Yes	Yes
15	Epée E et al. 2017^4^ (case 1, Father)	Yes	NR	Yes	Yes	Yes	NR	NR	Yes	Yes	Yes	Yes
16	Epée E et al. 2017^4^ (case 2, Daughter)	Yes	NR	Yes	Yes	Yes	NR	NR	Yes	Yes	Yes	Yes
17	Epée E et al. 2017^4^ (case 3,Grand Daughter)	Yes	NR	Yes	Yes	Yes	NR	NR	Yes	Yes	Yes	Yes

NR-Not reported.

Frontal bossing was another commonly observed feature, appearing in many of the included reports, reflecting underlying craniofacial dysmorphogenesis. Dental anomalies were widely documented, ranging from malocclusion and irregular dentition to more complex findings such as enamel defects and high-arched palate, particularly in the detailed dental assessments. Only a minority of cases reported enamel fragility or high-arched palate, indicating these features may be present but not consistently assessed.

Microstomia and retrognathia appeared with variable frequency, with retrognathia more prominently noted in the cases described by Jimenez-Armijo et al.^5^, Ammar et al.^6^, and all three members of the familial series from Epée et al.^4^ Hypotrichosis or alopecia was reported in several cases, aligning with the known ectodermal involvement in HSS. Older cases (e.g., Neki A et al.,^7^ Jain V et al.^8^) and familial cases (Epée E et al.^4^) also showed strong representation of these ectodermal findings.

**Fig. 2:**
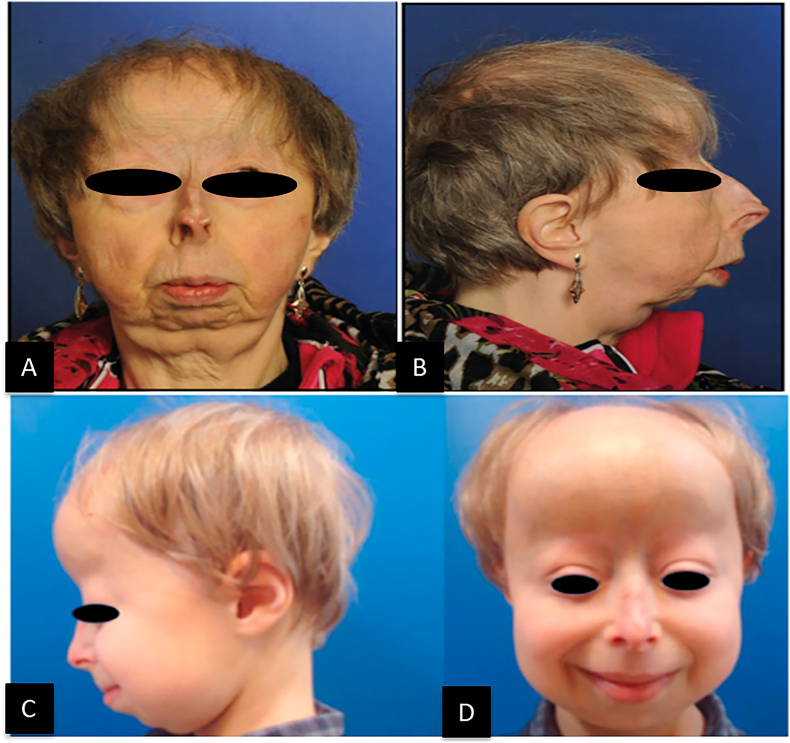
(A & B) Craniofacial features of an elderly female patient demonstrating a beaked nasal profile, micrognathia, and associated dysmorphic characteristics. Figure adapted from Sondeijker CFW, Apperloo RC, Kalaykova SI, Baan F, Maertens JKM. Combined orthodontic and surgical treatment for a patient with Hallermann-Streiff-Francois syndrome, severe obstructive sleep apnea, and history of antiresorptive medication. Am J Orthod Dentofacial Orthop. 2021; 159(1):97–107. *(C & D)* Craniofacial appearance of a 10-year-old patient showing frontal bossing, frontal skin atrophy with prominent veins, atrophic nasal skin with ulceration scars from spectacle pressure, a pinched thin nose, absence of eyelashes and eyebrows, and receding chin with mandibular hypoplasia. Figure adapted from Preudhomme R, Veyssiere A, Ambroise B, Benateau H. Hallermann-Streiff syndrome: Cranio-facial manifestations systematic review and report of two cases. J Stomatol Oral Maxillofac Surg. 2022; 123(4):e219-e223.

Micrognathia, mandibular hypoplasia, pinched nose, bird-like facies, dental anomalies, and frontal bossing forming the dominant clinical profile across published reports (Fig. 2).

### 3.4 Ophthalmologic features

Table 3presents the spectrum of ophthalmologic abnormalities reported across published case reports of Hallermann–Streiff syndrome. Ophthalmologic abnormalities were among the most consistently reported clinical features across the included cases of Hallermann–Streiff syndrome. Microphthalmia was highly prevalent, appearing in a majority of reports, often bilaterally or described as severe, as in cases reported by Abdulghafor M et al.^9^ and Epée E et al.^4^ Congenital cataracts were one of the hallmark findings, documented in almost all cases with available data, frequently necessitating early surgical intervention. Strabismus was also frequently identified, particularly in pediatric cases, reflecting early-onset ocular misalignment associated with the syndrome.

**Table 3:** Ophthalmological features reported across the included case reports of Hallermann–Streiff syndrome.

S.no	Author	Microphthalmia	Congenital cataracts	Strabismus	Blue sclera	Lack of eyelashes/eyebrows	Nystagmus	Microcornea	Shallow anterior chamber	Visual impairment	Glaucoma
1	Sondeijker C.F.W et al. 2021^13^	NR	NR	NR	NR	NR	NR	NR	NR	NR	NR
2	Preudhomme R. et al. 2022^10^ (Case 1)	Yes	Yes	Yes	Yes	Yes	No	No	No	No	No
3	Preudhomme R. et al. 2022^10^ (Case 2)	Yes	Yes	Yes	No	Yes	No	No	No	No	No
4	Abdulghafor M. et al. 2024^9^	Severe	Yes	No	No	No	No	Yes	Yes	No	No
5	Robinow M et al. 1991^16^	Yes	Yes	No	No	No	No	No	No	No	No
6	Pereira de Godoy AC et al. 2022^17^	NR	Yes	No	No	No	No	No	No	No	No
7	Jimenez-Armijo A. et al. 2021^5^	Bilateral	Yes	Yes	No	Yes	Yes	No	No	No	No
8	Shimada A. et al. 2022^15^	No	Yes	No	Yes	No	No	No	Yes	Yes	Yes
9	Guerin S. et al. 2022^14^	No	Yes	No	No	No	No	No	No	Yes	No
10	Agrawal P et al. 2021^3^	No	Surgery done	No	No	No	No	No	No	No	No
11	Neki AS 1993^7^	Yes	No	Yes	Yes	Yes	Yes	Yes	No	Yes	No
12	Jain V et al., 2011^8^	Yes	No	NR	Yes	Yes	Yes	Yes	NR	Yes	Yes
13	Ammar N et al. 2022^6^	Yes	No	Yes	NR	Yes	NR	NR	NR	NR	NR
14	Lee MC et al. 2008^11^	Yes	Yes	Yes	NR	Yes	Yes	Yes	NR	Yes	No
15	Epée E et al. 2017^4^ (case 1, Father)	Yes	Yes	Yes	NR	NR	Yes	Yes	No	Yes	NR
16	Epée E et al. 2017^4^ (case 2, Daughter)	Yes	Yes	Yes	NR	NR	Yes	Yes	NR	Yes	No
17	Epée E et al. 2017^4^ (case 3,Grand Daughter)	Yes	Yes	Yes	NR	NR	Yes	Yes	NR	Yes	No

NR-Not reported.

Features related to ectodermal involvement, such as sparse eyelashes or eyebrows, were variably reported but present in several cases, including those by Preudhomme et al.^10^, Jimenez-Armijo et al.^5^, Neki A et al.,^7^ Jain V et al.,^8^ and Lee MC et al.^11^ Blue sclera, although a classic component of the syndrome, appeared inconsistently across reports, being explicitly mentioned in only a small number of cases.

Nystagmus was noted in multiple instances, particularly in the familial cases reported by Epée E et al.^4^, as well as in cases from Jimenez-Armijo et al.^5^ and Jain V et al.,^8^ indicating a potential association with microphthalmia or congenital lens abnormalities. Microcornea appeared in several reports, most notably in cases with severe globe size reduction. Shallow anterior chamber, while less commonly documented, was reported in some surgically managed cases.

Visual impairment was common across all age groups, typically secondary to congenital cataracts, microphthalmia, or postoperative aphakia. Glaucoma was rarely described, with only isolated reports indicating suspected or confirmed elevated intraocular pressure.

Collectively, these findings underscore the prominent ophthalmologic burden in Hallermann–Streiff syndrome and support the need for early, comprehensive, and on-going ophthalmologic evaluation as a core component of patient management.

David LR et al.^12^ (1999) reported a unique series of 15 patients with Hallermann–Streiff syndrome, representing the largest single cohort in the available literature (Table 4). All patients demonstrated dyscephaly, reinforcing its status as a universal hallmark of the syndrome. Dental abnormalities were also highly prevalent, ranging from small or missing teeth to complete anodontia in several individuals. Ocular involvement was prominent, with most patients exhibiting microphthalmia, congenital cataracts, or both.

**Table 4:** Clinical Characteristics of Patients with Hallermann–Streiff Syndrome Reported by David LR et al.,^12^ 1999. (n = 15).

Patient No.	Sex	Dyscephaly	Dental Abnormalities	Microphthalmia	Cataracts	Skin Atypia	Short Stature	Hypotrichosis	Mental Retardation	Cardiac Problems	Respiratory Problems	Sleep Apnea	Offspring
1	M	Y	Small teeth	Y	Y	Y	Y	Y	Mild	N	N	Y	N
2	F	Y	Missing teeth	N	Y	N	Y	Y	Mild	Y	Y	N	N
3	M	Y	Small	N	Y	N	N	Y	Severe	N	Y	Y	N
4	M	Y	Minor	Y	Y	N	Y	Y	—	N	N	Y	N
5	M	Y	Y	Y	—	Y	Y	Y	—	N	—	N	—
6	F	Y	Small	Y	—	Y	Y	Y	Severe	Y	Y	Y	N
7	M	Y	No teeth	Y	Y	—	Y	Y	—	Y	Y	Y	1 Normal
8	F	Y	Small	Y	Y	Y	Y	Y	Severe	Y	Y	Y	—
9	M	Y	Y	Y	Y	Y	Y	Y	—	Y	Y	Y	N
10	F	Y	Minor	Y	Y	Y	Y	Y	Mild	—	—	N	1 Normal
11	F	Y	Minor	Y	Y	Y	Y	Y	—	Y	Y	—	2 Normal
12	F	Y	Y	Y	Y	Y	Y	Y	—	N	N	—	1 Normal
13	F	Y	Y	Y	Y	Y	Y	Y	—	N	N	N	—
14	F	Y	Y	Y	Y	Y	Y	Y	—	—	—	N	—
15	M	Y	Missing teeth	Y	Y	Y	Y	Y	—	N	N	N	—

M-male, F- Female, Y-Yes, N-No.

**Table 5:** Risk of bias assessment.

S.no	Author	Q1	Q2	Q3	Q4	Q5	Q6	Q7	Q8	Percentage	Risk of bias
1.	Sondeijker C.F.W. et al., 2021	Yes	Yes	Yes	Yes	Yes	Yes	Yes	Yes	100%	Low
2.	Preudhomme R et al., 2022 (2cases)	Yes	No	Yes	No	Yes	No	No	Yes	50%	Moderate
3.	Abdulghafor M et al., 2024	Yes	Yes	Yes	Yes	Yes	Yes	Yes	Yes	100%	Low
4.	Robinow M et al., 1991	Yes	Yes	Yes	Yes	Yes	Yes	Yes	Yes	100%	Low
5.	Godoy ACPD et al., 2022	Yes	Yes	Yes	Yes	Yes	Yes	No	Yes	87.5%	Low
6.	Agrawal P et al., 2021	Yes	Yes	Yes	Yes	Yes	Yes	Yes	Yes	100%	Low
7.	Jimenez-Armijo et al., 2021	Yes	Yes	Yes	Yes	Yes	Yes	No	Yes	87.5%	Low
8.	Guerin S et al., 2022	Yes	Yes	Yes	Yes	Yes	Yes	Yes	Yes	100%	Low
9.	Shimada A et al., 2022	Yes	Yes	Yes	Yes	Yes	Yes	Yes	Yes	100%	Low
10.	Neki AS., 1993	Yes	Yes	Yes	Yes	No	No	No	Yes	62.5%	Moderate
11.	David LR et al., 1999 (15 cases)	Yes	Yes	Yes	Yes	No	No	No	Yes	62.5%	Moderate
12.	Jain V et al., 2011	Yes	Yes	Yes	Yes	Yes	Yes	Yes	Yes	100%	Low
13.	Ammar N et al., 2022	Yes	Yes	Yes	Yes	Yes	Yes	Yes	Yes	100%	Low
14.	Lee MC et al., 2008	Yes	No	Yes	No	Yes	No	No	Yes	50%	Moderate
15.	Epée E et al., 2017 (3 Cases)	Yes	Yes	Yes	Yes	Yes	Yes	Yes	Yes	100%	Low

JBI checklist consists of 8 questions, each scored as Yes or No.

1. Were patient's demographic characteristics clearly described?.

2. Was the patient's history clearly described and presented as a timeline?.

3. Was the current clinical condition of the patient on presentation clearly described?.

4. Were diagnostic tests or assessment methods and the results clearly described?.

5. Was the intervention(s) or treatment procedure(s) clearly described?.

6. Was the post-intervention clinical condition clearly described?.

7. Were adverse events (harms) or unanticipated events identified and described?.

8. Does the case report provide takeaway lessons?.

Additional common features included skin atrophy, hypotrichosis, and short stature, each reported in a substantial proportion of cases. The severity of intellectual impairment varied, with some individuals showing mild to severe mental retardation, while others demonstrated no cognitive deficits.

Respiratory issues and sleep apnoea were noted in many patients, highlighting the syndrome's potential for significant airway compromise. Cardiac problems were reported in a smaller subset. Among the adult patients with documented reproductive history, several had normal offspring, indicating that fertility may be preserved despite multisystem involvement.

Overall, this cohort highlights the broad phenotypic spectrum of Hallermann–Streiff syndrome, with consistent craniofacial and dental features, frequent ocular pathology, and variable systemic manifestations.

### 3.5 Risk of bias reporting

Risk of bias was evaluated using the Joanna Briggs Institute (JBI) Critical Appraisal Checklist for Case Reports, which includes eight domains addressing the clarity and completeness of patient demographics, history, clinical presentation, diagnostic assessment, interventions, post-intervention course, adverse events, and key lessons.

Overall, the methodological quality of the included studies was moderate to high. The majority of reports (10 out of 15) achieved a low risk of bias, scoring between 87.5% and 100%, reflecting comprehensive reporting of patient characteristics, diagnostic findings, and management outcomes. Several recent case reports—including those by Sondeijker CFW et al.^13^, Abdulghafor M et al.^9^, Guerin S et al.^14^, Shimada A et al.^15^, and Jain V et al.^8^—demonstrated excellent compliance with all eight JBI domains.

A smaller subset of studies (five reports) exhibited moderate risk of bias, predominantly due to incomplete reporting of patient history, adverse events, or post-intervention outcomes. Notably, earlier publications such as those by Neki A et al.,^7^ and David LR et al.^12^ lacked detailed descriptions of post-treatment progression and adverse events. Similarly Preudhomme et al.^10^, and Lee MC et al.^11^ showed partial reporting across several items, resulting in lower overall scores (50–62.5%).

Despite these variations, all included cases provided sufficient clinical detail to contribute meaningfully to the synthesis. However, the variability in reporting underscores the importance of standardized case report guidelines to improve transparency and reproducibility in rare-disease literature.

## 4 Discussion

This study represents the first comprehensive synthesis integrating airway, craniofacial, and ophthalmologic manifestations of Hallermann–Streiff syndrome. It consolidates evidence from published case reports to delineate the clinical spectrum, airway-related challenges, ophthalmologic abnormalities, and management approaches in patients with Hallermann–Streiff syndrome. Across the included cases, several characteristic features consistently emerged, including micrognathia, mandibular hypoplasia, nasal abnormalities, dental anomalies, congenital cataracts, strabismus, microphthalmia, hypotrichosis, and blue sclera.^5^^,^^9^^,^^10^^,^^13^^,^^16^^,^^17^ Airway-related complications—most notably obstructive sleep apnoea (OSA), neonatal respiratory distress, nasal obstruction, and difficult intubation—were also frequently reported. Although the clinical manifestations varied in severity, the overall pattern supports the well-established multisystem involvement of HSS.

Airway compromise remains a major clinical concern in HSS. Several cases in this review described severe OSA, neonatal respiratory distress, requirement of non-invasive ventilation (NIV), and challenges in mask ventilation and endotracheal intubation. Structural contributors, such as micrognathia, retrognathia, nasal hypoplasia, and narrow nostrils, were consistently identified.

Management strategies included mandibular advancement, orthognathic surgery, CPAP/BiPAP, adenoidectomy, and in severe cases, tracheostomy. Long-term NIV showed favourable outcomes in infants.^14^ These findings highlight the importance of early respiratory evaluation, sleep studies, and multidisciplinary airway assessment.

The review suggests that craniofacial anomalies are a prominent and characteristic clinical feature of HSS. Features such as micrognathia, retrognathia, pinched nose, dental malformations, and high arched palate appeared frequently. Dental anomalies were reported in nearly all cases, ranging from microdontia to enamel fragility and missing teeth, suggesting the need for on-going dental surveillance from early childhood.^5^^,^^9^^,^^16^^,^^17^

Craniofacial deformities occasionally progressed into functional issues, including feeding difficulties, recurrent aspiration, and challenges with oral hygiene. A few reports also documented successful correction using orthognathic procedures,^13^ facial lipofilling,^10^ and lymphatic therapy^17^ for nasal obstruction.

Ophthalmologic involvement was universal among reported patients, although the type and severity varied. The most common findings were congenital cataracts, microphthalmia, strabismus, blue sclera, persistent pupillary membranes, microcornea, and significant visual impairment.^4^^,^^5^^,^^7^^,^^8^^,^^10^ Early cataract extraction, where feasible, showed meaningful improvement in visual function.

Late diagnosis—as in older adults—still revealed characteristic ocular signs that aided retrospective clinical identification of HSS. These findings emphasize ophthalmologic evaluation as a central component of diagnostic workup.

Although HSS has well-described phenotypic traits, this review observed substantial heterogeneity in the extent and severity of craniofacial, airway, and ocular involvement. Some adults presented without significant airway symptoms,^4^^,^^7^^,^^15^ while infants frequently exhibited severe respiratory distress.^6^^,^^9^^,^^16^

This variability suggests a spectrum-like expression of the syndrome that may be influenced by genetic, environmental, or developmental factors, though the exact etiology remains unknown. An interesting highlight of this review is the three-generation familial cluster reported by Epée E et al.^4^, involving a father, his daughter, and granddaughter—all demonstrating classic Hallermann–Streiff craniofacial and ophthalmologic features with remarkable consistency. This series underscores the possibility of hereditary transmission in at least a subset of cases, despite the condition being generally considered sporadic. This familial pattern may be described as three successive generations exhibiting similar phenotypic features, highlighting that even in rare disorders, hereditary influences can play a subtle yet significant role. Based on this review, several recommendations emerge for optimal management of Hallermann–Streiff syndrome. Routine airway evaluation, including early sleep studies, is essential to identify and manage obstructive sleep apnoea at the earliest stage. Ophthalmologic screening should also be initiated early in life to detect congenital cataracts and prevent long-term visual impairment such as amblyopia. Effective care for these patients requires a coordinated multidisciplinary approach involving ENT specialists, ophthalmologists, paediatricians, dental surgeons, and craniofacial teams. Non-invasive ventilation should be considered a first-line intervention for infants presenting with obstructive sleep apnoea, given its reported success in improving respiratory outcomes. Additionally, long-term follow-up is crucial, as many complications may be progressive or may manifest later in life.

## 5 Limitations & future directions

This review is inherently limited by the nature of case reports, including non-standardized reporting, potential publication bias favouring severe or unusual presentations, the inability to perform quantitative synthesis, and the frequent lack of long-term follow-up. Additionally, the descriptive aggregation of data and heterogeneity across cases introduce variability that may influence the reported distributions, and the absence of confidence intervals further limits the precision of these estimates. In addition, a number of case reports are published in non-indexed or regional journals that were not retrievable**,** which may have led to the omission of relevant cases and limited the comprehensiveness of this review. Despite these limitations, the collective findings still offer valuable insights into the rare but clinically meaningful patterns associated with Hallermann–Streiff syndrome. Future research should focus on establishing standardized reporting guidelines for HSS, developing prospective multicenter registries to support more robust data analysis, conducting genetic studies to better understand underlying etiological mechanisms, and ensuring longitudinal follow-up to clarify the natural history and progression of the condition.

## 6 Conclusion

This review consolidates the current understanding of the multisystem involvement in Hallermann–Streiff syndrome. Despite phenotypic variability, consistent patterns across airway, craniofacial, dental, and ophthalmologic systems were observed. Early diagnosis, proactive airway management, and coordinated multidisciplinary care remain central to improving patient outcomes.

## Human and animal statements

This study is a systematic review of published literature and did not involve direct participation of human subjects or animals. Therefore, ethical approval and informed consent were not required.

## Declaration of competing interest

The authors declare that they have no known competing financial interests or personal relationships that could have appeared to influence the work reported in this paper.
